# Acute Respiratory Failure, Ischemic Modifications on Electrocardiogram: Alternative Etiology—A Case of Morgagni Hernia

**DOI:** 10.3390/medicina58020204

**Published:** 2022-01-28

**Authors:** Agnes Zsuzsánna Szász, Enikő Dalma Székely-Vass, Gyopár Tunde Lovász, Annamária Magdás, Loránd Jozsef Ferencz

**Affiliations:** 1 “George Emil Palade” University of Medicine, Pharmacy, Science and Technology, 540142 Târgu Mureș, Romania; zsuzsanna.szasz@umfst.ro (A.Z.S.); anamaria.magdas@umfst.ro (A.M.); lorand.ferencz@umfst.ro (L.J.F.); 2 Internal Medicine Department I, Târgu Mureș County Emergency Clinical Hospital, 540136 Târgu Mureș, Romania; 3 Occupational Medicine Department, Mureș County Hospital, 540072 Târgu Mureș, Romania; 4 Internal Medicine Department III, Mureș County Hospital, 540072 Târgu Mureș, Romania

**Keywords:** Morgagni hernia, acute respiratory failure, chest discomfort, mimicking ischemia via electrocardiography (ECG), chest computer tomography (CT)

## Abstract

We discovered a rare pathology described in adulthood, followed by the development of a long asymptomatic evolution, which underlined the importance of multidisciplinary collaboration. We present the case of a 62-year-old female smoker patient, with a known previous medical history of chronic ischemic heart disease, hypertension, chronic obstructive pulmonary disease (COPD), gastric ulcer and gastritis. The patient was rushed to the emergency room (ER) with acute respiratory failure, chest discomfort, ankle and facial edema and a chest X-ray showing a right lower pulmonary lobe consolidation, with an alarming ischemic electrocardiogram (ECG) modification without increasing myocardial cytolysis indicators. This led our medical team to investigate a possible cardiovascular event that might have been in development. After immediate admission, thoracic computer tomography (CT) imaging was carried out, which found a Morgagni diaphragmatic hernia, containing adipose tissue and the hepatic flexure of the colon with approximate dimensions of 50/100 mm. We faced differential diagnostic problems. We knew the subject’s existing cardiac and chronic respiratory tract pathologies from their previous medical history; therefore, multiple investigations and check-ups were carried out. A chest CT and surgery intervention were needed to resolve this case. Subsequently, the acute respiratory failure and alarming ischemic ECG modification disappeared.

## 1. Introduction

The Morgagni hernia is a rare anterior defect of the diaphragm (retrosternal or parasternal hernia), characterized by the herniation through the foramina “Morgagni” or sternocostal triangle, resulting from the incomplete fusion of the septum transversum and sternum [1].

This malformation was first described in 1769 as an anatomical defect that allows the abdominal organs to penetrate into the thorax [2], leading to impaired lung function and other circulatory consequences.

The Morgagni hernia accounts for approximately 1.5% of all congenital diaphragm hernia (CDH) cases [2,3]. In most cases, Morgagni hernias are diagnosed late due to the fact that patients can be asymptomatic or can display vague gastrointestinal and respiratory signs and symptoms.

A study performed in 2008, which involved 298 patients diagnosed with a Morgagni hernia between 1951 and 2007 found out that the most common symptoms were pulmonary complaints (36%), which affected males more frequently and was characterized by early onset [4].

The pathophysiology of diaphragmatic hernias is not clear, since patients with previous normal chest X-ray images led clinicians to the following conclusion: this type of hernia may be acquired through a congenital defect in the diaphragm, and the clinical signs could delay an early diagnosis due to confusion with a pulmonary pathology, such as pneumonia [2]. Thus far, ultrasonography has been shown to be a useful diagnostic tool in detecting diaphragm hernias, but computer tomography (CT) seems to be the most sensitive method, as it describes not only the content of the hernia but also its complications [5].

Therefore, the aim of this case report was to highlight the characteristics of a Morgagni hernia and to warn clinicians about confusion with a pulmonary disease, which could lead to an incorrect diagnosis.

## 2. Case Presentation

We present the case of a 62-year-old smoker female patient, with a medical history of chronic obstructive pulmonary disease (COPD), chronic ischemic heart disease, hypertension, gastric ulcer, gastritis and diaphragmatic hernia containing adipose tissues (described at a previous CT-angiography that was performed to explain the etiology of the posterior thoracic and epigastric pain, which was thought to be due to coronary artery disease).

At the time of presentation to the emergency room (ER), the patient’s complaints were dyspnea on minimal exertion, and chest discomfort with an onset of approximately 10 days. Previously, the patient was consulted by the regional internal medicine specialist, who prescribed a topical treatment containing long-acting beta 2 agonist and corticosteroids, as well as prednisone, but without any improvement.

From the patient’s personal medical history, we must note that during the last 3 years, she has presented an intermittent tearing epigastric pain with irradiation in the interscapular spinal region several times. Therefore, the patient underwent multiple examinations, which did not show any significant pathological changes, except erosive prepyloric antral gastritis with the presence of *Helicobacter pylori*. After the recommended treatment, the intermittent epigastric pain reappeared and was still present from time to time.

To exclude a cardiac component of the symptoms, an electrocardiogram (ECG), a cardiac ultrasound, was performed, which found borderline left ventricular hypertrophy, with normal left ventricular systolic function, diastolic dysfunction (impaired relaxation pattern) and minimal aortic sclerosis. A cardiac stress test was also performed, which showed a negative result, and coronary computed tomography angiography (CCTA) revealed multiple coronary artery plaques with stenosis atheromatous up to a maximum of 50 % and a diaphragmatic hernia with adipose tissues.

**Figure 1:**
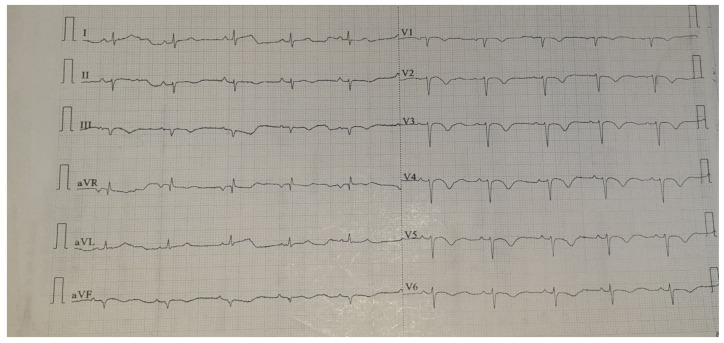
ECG with negative T-waves in the precordial leads.

**Figure 2:**
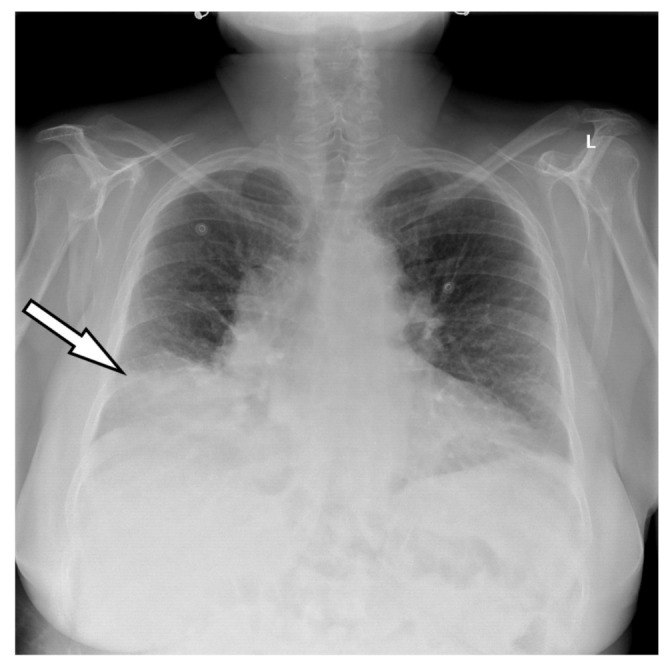
Posteroanterior chest X-ray: a right lower pulmonary lobe consolidation.

When the patient was at the emergency room, during the examination, she was found with anterior thoracic and abdominal discomfort; mild fever; an oxygen saturation of around 85%; mild leg and facial edema; diminished respiratory sounds above the right lung area; faded cardiac sounds; a blood pressure of 130/82 mmHg; 70 beats/min heart rhythm; and a distended abdomen, without any pain during palpation. The laboratory tests showed elevated leukocyte and neutrophil levels. The biological markers characteristic of an acute myocardial lesion were negative. However, the ECG ischemic changes were described with negative T-waves in the precordial leads (Figure 1). These modifications were not described upon the previous ECG. A chest X-ray was performed at the ER, showing a right lower pulmonary lobe consolidation (Figure 2).

Repeated cardiologic check-ups were performed, followed by a cardiac ultrasound in the ER without new alterations in the left ventricular wall motion, and the *ejection fraction* was *60% without any* free pericardial space. The patient was admitted to the internal medicine department for pneumonia treatment.

**Figure 3:**
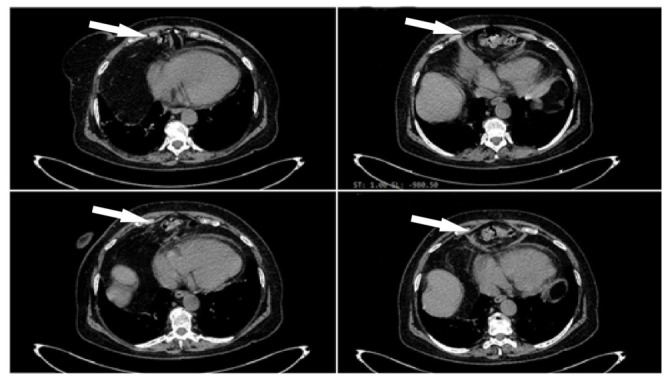
Chest CT imaging: right anterior diaphragmatic hernia, containing adipose tissues and the hepatic flexure of the colon.

Thanks to the consolidation seen on the chest X-ray in order to exclude a lung tumor, a chest CT was carried out in the first 24 hours. The result of the test showed a 37 mm herniation through the foramina “Morgagni”, and contained adipose tissues, as well as a hepatic flexure of the colon with approximate dimensions of 50/100 mm (Figure 3).

Due to the persistence of the abdominal and respiratory symptoms, the dyspnea did not improve, there was an absence of intestinal transit for stool, and the result of the CT examination showed the possibility of infarction or the strangulation of the colon's herniated segment. The patient was transferred to the surgical department for immediate surgical treatment.

**Figure 4:**
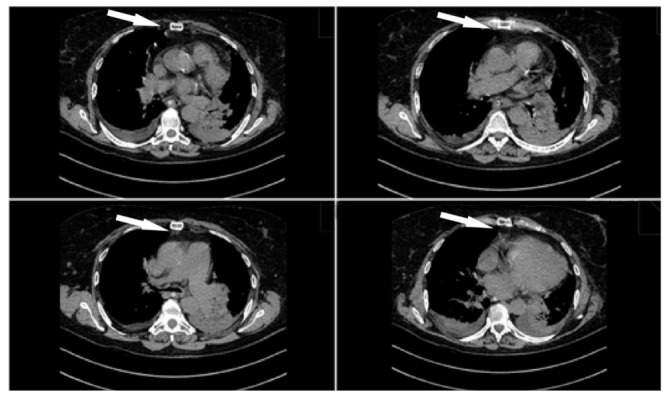
Postoperative chest CT imaging: closed diaphragmatic hernia.

An upper midline laparotomy was performed, the hernial sac was excised and the diaphragmatic defect was closed with some interrupted sutures (Figure 4).

After the 1st postoperative day, the patient became unconscious; the arterial gasometry revealed hypercapnic coma, invasive mechanical ventilation was required in order to support and monitor her vital functions, and an immediate broad-spectrum antibiotic treatment was started. Several bronchial aspirates were performed, and a full septic screen was repeated—all of them came back negative. The laboratory data showed leukocytosis with neutrophilia. After the chest X-ray and the CT were repeated and showed no significant changes, we concluded that the coma was the result of the COPD exacerbation.

**Figure 5:**
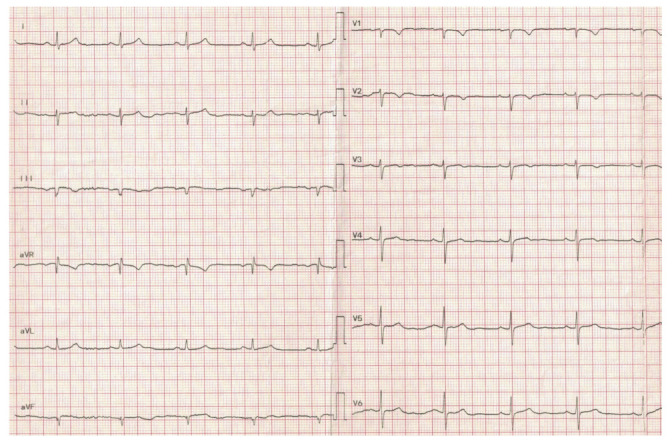
Normal ECG—negative left precordial T-waves disappeared.

Thanks to the intensive care unit and the treatment she had received, the patient recovered quickly from the unconscious state, and she was discharged on the 12th postoperative day. The ECG modification—negative T-waves in the left precordial leads—disappeared during the convalescence period (Figure 5).

## 3. Discussion

While some patients with this type of hernia may present chest pain, epigastric pain or signs of intestinal obstruction [6], most of them remain asymptomatic, and a hernia could be detected incidentally during a chest X-ray or other investigations. A similar asymptomatic case was discussed in an article in 2015, where a 42-year-old male patient who presented with acute coronary syndrome required immediate coronary artery bypass surgery and was found with a giant Morgagni hernia [7]. The question is, what is considered to be asymptomatic? This is due to the fact that the most minor modification of oxygen saturation can have a huge impact on medical decision making [8,9].

Another case published in 2013 described a young female patient with a chest X-ray characteristic of cardiomegaly with normal cardiac biomarkers and a T-wave inversion in the V1–V5 leads, but the cardiac MRI revealed a mass of herniation throughout the foramen of the Morgagni. In rare cases, they can be symptomatic, as in our case, where the patient complained of intermittent chest pain and shortness of breath on exertion [10]. The most recently debated case in the *Canadian Journal of Cardiology* presents a case of an elderly female patient who complained of dyspnea on exertion; showed T-wave inversion upon ECG, a coronary angiography negative for ischemia; and was diagnosed with diaphragmatic hernia with cardiac dextroposition [11]. The ECG-specific abnormality in healthy women with T-wave inversion in precordial leads is very suspicious of ischemia, but there are very few cases in the medical literature of these modifications being related to a Morgagni hernia [10,12]. Zanini’s medical team proposed that ECG modifications can be associated with hiatal hernia [13].

Very similar to our case, some ECG modifications can be related to cardiac compression by a hiatal mass and can lead to misdiagnosis from cardiomegaly to acute coronary syndrome. In all these cases, the truth was beyond a simple cardiovascular pathology, although the hypothesized methods—a chest X-ray and an electrocardiogram—and our symptomatology, led the medical team to a cardiovascular event.

In our case, the computer tomography imaging was the chosen method [14] for demonstrating the presence of omentum fat, and the intestinal air was located outside its usual location.

In many countries, CT is used as first-line imaging in the case of a chest pain or persistent epigastric pain. A Morgagni hernia should be considered as a potential diagnosis in the case of acute respiratory failure, intermittent abdominal pain and chest X-ray modifications [15]. The medical literature emphasizes that sometimes a simple chest X-ray is not stimulating enough for this kind of pathology, as the overexposed picture can lead to the diagnosis of a different pathology, as in our case [16]. Thus far, there have been cases in which the presence of recurrent lower respiratory tract infections caused by the simple presence of Morgagni hernia [17] were described.

In many cases, less importance is assigned to diaphragmatic hernias with fat content^1^ compared to the presence of recurrent lower respiratory tract infections caused by the simple presence of Morgagni hernia [18].

As described in our case, the CT angiography first appeared one year earlier, but without any intestinal content. The process of simply visualizing and setting up a positive diagnosis was started by Soykan Arian and her medical team when analyzing 21 patients with Morgagni hernia. This study resulted in an interesting conclusion—dyspnea was the most prominent symptom [19], and computerized tomography was the gold standard for an accurate diagnosis [19,20], as in our case.

A surgical intervention is also indicated [21] for asymptomatic patients due to the danger of imminent bowel incarceration.

A study conducted in 2005, debates the fact that acute respiratory failure may occur after abdominal surgery, underlining its conclusion that postoperative pulmonary complications are considered to be a significant problem after upper abdominal surgery [22], which explains the presence of muscular deficiency and hypoventilation. The existing COPD was the factor that led to the worsening of the patient’s condition.

### Particularity of the Case

In our case, acute respiratory failure—oxygen saturation of 85%—intermittent epigastric pain and anterior thoracic discomfort, a low-grade fever, chest X-ray modifications and ECG changes with T-wave inversion made us consider the following signs: right lower pulmonary lobe pneumonia and a diffuse ischemic coronary lesion.

The chest CT concluded that all the symptoms were caused by a particular form of diaphragmatic hernia—Morgagni hernia. All these ECG changes—negative T-waves in the left precordial leads—disappeared in the convalescence period.

The heterogeneity of the signs and symptoms can be considered as a particularity in our case. In the medical literature, there are cases where only ECG modifications can lead to misdiagnosis, but here, we observed a mixture of signs and symptoms for both cardiovascular and pulmonary pathology—with signs of severe acute respiratory failure—which makes our case unique.

## 4. Conclusions

Once diagnosed, the requirement for surgery is critical; this surgical repair is important to avoid further complications. Emergency intervention is not always necessary, unless there is clear evidence of strangulation. Treatment consists of direct closure of the diaphragmatic defect, and suturing by transabdominal or transthoracic access.

All unknown abdominal, epigastric and thoracic pains with unknown etiology need to be properly investigated, even if all initial examinations show normal results—normal laboratory results, ultrasound, ECG, cardiologic and gastroenterological check-ups. Nevertheless, persistent pain can influence a person’s quality of life and general functioning, and the underlying cause may be much simpler than we think. In order to highlight special cases, there is a need for multidisciplinary collaboration.

## Data Availability

The data used in this manuscript can be found in the database of the Targu Mures, County Emergency Clinical Hospital, Mures, Romania.

## Abbreviations

The following abbreviations are used in this manuscript:

COPD	chronic obstructive pulmonary disease
ER	emergency room
ECG:	electrocardiogram
CT	computer tomography
CDH	congenital diaphragm hernia
CCTA	coronary computed tomography angiography
